# Month 2 Culture Status and Treatment Duration as Predictors of Recurrence in Pulmonary Tuberculosis: Model Validation and Update

**DOI:** 10.1371/journal.pone.0125403

**Published:** 2015-04-29

**Authors:** Robert S. Wallis, Thomas Peppard, David Hermann

**Affiliations:** 1 Aurum Institute, Johannesburg, South Africa; 2 Certara, LP, St. Louis, Missouri, United States of America; National Institute of Allergy and Infectious Disease, UNITED STATES

## Abstract

**Background:**

New regimens capable of shortening tuberculosis treatment without increasing the risk of recurrence are urgently needed. A 2013 meta-regression analysis, using data from trials published from 1973 to 1997 involving 7793 patients, identified 2-month sputum culture status and treatment duration as independent predictors of recurrence. The resulting model predicted that if a new 4-month regimen reduced the proportion of patients positive at month 2 to 1%, it would reduce to 10% the risk of a relapse rate >10% in a trial with 680 subjects per arm. The 1% target was far lower than anticipated.

**Methods:**

Data from the 8 arms of 3 recent unsuccessful phase 3 treatment-shortening trials of fluoroquinolone-substituted regimens (REMox, OFLOTUB, and RIFAQUIN) were used to assess and refine the accuracy of the 2013 meta-regression model. The updated model was then tested using data from a treatment shortening trial reported in 2009 by Johnson et al.

**Findings:**

The proportions of patients with recurrence as predicted by the 2013 model were highly correlated with observed proportions as reported in the literature (R2 = 0.86). Using the previously proposed threshold of 10% recurrences as the maximum likely considered acceptable by tuberculosis control programs, the original model correctly identified all 4 six-month regimens as satisfactory, and 3 of 4 four-month regimens as unsatisfactory (sensitivity = 100%, specificity = 75%, PPV = 80%, and NPV = 100%). A revision of the regression model based on the full dataset of 66 regimens and 11181 patients resulted in only minimal changes to its predictions. A test of the revised model using data from the treatment shortening trial of Johnson et al found the reported relapse rates in both arms to be consistent with predictions.

**Interpretation:**

Meta-regression modeling of recurrence based on month 2 culture status and regimen duration can inform the design of future phase 3 tuberculosis clinical trials.

## Introduction

Tuberculosis remains one of the world’s deadliest communicable diseases, causing an estimated 9 million new cases and 1.5 million deaths annually [1]. The identification of new regimens capable of shortening treatment without increasing the risk of recurrence has been a high priority for tuberculosis research for many years. A brief report by Mitchison in 1993 first proposed a role for sputum culture status after 2 months of treatment in the evaluation of such regimens [2]. Two subsequent independent analyses of regimen pairs of equal duration confirmed the relationship between sputum culture status and relapse risk [3,4]. However, the design of these studies precluded their ability to directly inform the likelihood of success of shorter new regimens in phase 3 trials.

In 2013, a meta-regression analysis identified 2-month sputum culture status and treatment duration as independent predictors of recurrence, using data from 7793 patients treated with 58 diverse regimens of various durations published from 1973 to 1997 [5]. The regression model predicted that if a new 4-month regimen reduced the proportion of patients positive after 2 months of treatment to 1%, it would reduce to 10% the risk of a relapse rate >10% in a trial with 680 subjects per arm. The 1% target was far lower than anticipated.

There have since been lingering concerns that the model, which was developed using data from decades-old trials, might have limited ability to predict results of contemporary studies. In October 2014, results of 3 phase 3 trials of 4 fluoroquinolone-substituted 4-month regimens were reported [6–8]. None of the four 4-month regimens tested in these trials proved successful. In the present publication, data from these trials have been used to assess and refine the accuracy of the 2013 meta-regression model. The accuracy of the updated model was then assessed using data from the treatment shortening study of Johnson *et al* [9]. None of these studies had been included during development of the original model.

## Methods

### Model validation

The original dataset, statistical programming code, and resulting mathematical model, as reported in 2013, comprised the training set for this study. That model predicted TB recurrence risk based on the proportion positive at month 2 and the treatment duration in months, as follows: logit(recurrence proportion) = 2.1471 + 0.4756 x logit(month 2 positive proportion)- 2.2670 x ln(months duration). Proportions (recurrence and positive cultures at month 2) were transformed using the logit function. On an ordinary scale such proportions must be between 0 and 1. After logit transformation, values range from negative infinity to positive infinity, with logit(0.5) = 0. Logit transformation eliminates the possibility that a linear model will yield predicted proportions exceeding the limits of 0 and 1. Duration was transformed using the natural log function.

The validation dataset consisted of results from the REMox, OFLOTUB, and RIFAQUIN studies [6–8]. For consistency with historic data, recurrence rates were calculated from those studies as the number of recurrences divided by the number of subjects at risk for recurrence (*i*.*e*., excluding those who had unsatisfactory outcomes prior to being assessed for recurrence), as reported in per-protocol analyses. The REMox and RIFAQUIN trials included in their primary analyses patients retreated for recurrent tuberculosis based on clinical criteria without full microbiologic confirmation (described in the two studies as “retreated” and “limited bacteriology” cases, respectively). For consistency, these cases are included in the primary analysis in the present study as they were reported; a secondary analysis includes only those with full culture confirmation. Sputum culture status (positive or negative for *M*. *tuberculosis*) after 2 months of treatment is as reported in each trial using solid culture medium, excluding invalid results due to contaminated or missing specimens (REMox supplemental table S8, OFLOTUB table 2, RIFAQUIN supplemental table 2). Proportions positive for *M*. *tuberculosis* at this single time point (without regard to subsequent cultures) were used for consistency with historic data. The confidence intervals of observed proportions were estimated using logistic regression and the Wald test [10]. Validation of the model was performed by examining the relationship between observed and predicted recurrence proportions on a logit scale.

### Model updating

After the validation step, the model was updated using the full dataset, following the same methods as in the 2013 publication. Briefly, proportions were transformed using the logit function. Proportions reported as zero were assigned values of 0.005 (0.5%). As in the 2013 publication, the model included fixed effects for the logit of the month 2 culture positive rate and for the natural logarithm of the treatment duration. A random intercept was included for study. The within-study variance of each study arm was fixed using the asymptotic variance of the logit-transformed recurrence proportion, calculated as 1/Np(1-p), where N was the arm’s sample size and p was the recurrence proportion. The between-study variance was estimated by restricted maximum likelihood using the SAS MIXED procedure [11]. Regression parameters were estimated via weighted least squares using the inverse of the sum of the within-study variances as the weight. From the fitted model, we predicted recurrence proportions at given proportions of month 2 culture positivity and treatment duration. Two-tailed 80% confidence intervals (CI) were calculated, as well as corresponding prediction intervals (PI) for a hypothetical trial with 680 subjects per arm. The upper limit of this interval thus identifies the recurrence rate with only a 10% chance of being exceeded in a typical phase 3 trial (i.e., 90% power). The 10% value had been selected as the highest risk of failure likely to be considered acceptable by a pharma sponsor during the planning of such a trial. The prediction error variance on the logit scale was *SE* ^*2*^ *+ Vs + 1/N*_*new*_ *q*(1-*q*), where *q* was the model-predicted logit recurrence proportion at a given level of month 2 culture positive rate and treatment duration, *SE* was the standard error of *q*, *N*_*new*_ was the number of subjects per arm of the hypothetical trial, and *Vs* was the estimated variance associated with the study. The intervals were formed on the logit scale and back-transformed to an ordinary scale. The SAS code for the model is available on request.

## Results

Characteristics of the original (training) dataset as reported in 2013, the validation dataset (from REMox, OFLOTUB, and RIFAQUIN trials), and the full dataset are described in Table 1. The regimens are diverse with respect to their composition, duration, and region of the world in which they were studied. Relative to the original data set, the regimens in the validation set were shorter, included more subjects, were more likely to contain rifampin, pyrazinamide, and fluoroquinolones, and were more likely to have been conducted in Africa. These differences are expected, as they reflect advances in tuberculosis treatment and clinical trials over a period of nearly 4 decades.

**Table 1:** Characteristics of regimens included in the original (training), validation, and full dataset.

Characteristic	Dataset		
	Original	Validation	Full
Year of publication	1978 (1973–1980)	2014 (2014–2014)*	1979 (1973–1983)
Studies/regimens/subjects (N)	24/58/7793	3/8/3388	27/66/11181
Subjects per regimen	111 (75–163)	503 (169–635)*	117 (79–175)
Inclusion of rifampin and pyrazinamide	0.5 (0.5–1)	1 (1–1)*	0.5 (0.5–1)
Inclusion of fluoroquinolones	0 (0–0)	1 (0–1)*	0 (0–0)
Study conducted in Africa	0 (0–0.5)	1 (0.5–1)*	0 (0–1)
Proportion with recurrence	0.05 (0.02–0.08)	0.1 (0.04–0.15)	0.05 (0.03–0.08)
Proportion month 2 culture positive	0.13 (0.09–0.25)	0.13 (0.06–0.16)	0.13 (0.08–0.23)
Duration of treatment (months)	6 (6–6)	5 (4–6)*	6 (6–6)

Values indicate median (IQR) except as indicated. Regimens were scored as 1 if a criterion was fully met, 0.5 if partially met, and 0 if absent. Asterisks indicate differences between training and validation datasets at P≤.01 by Mann-Whitney rank test.

Detailed characteristics of the validation dataset from the 3 recent fluoroquinolone trials are described in Table 2. The numbers of patients with recurrences according to stringent and less-than-stringent criteria are shown as they were reported in the REMox and RIFAQUIN trials. The potential impact of recurrences without full microbiologic confirmation was greatest for the control arm of the REMox trial, in which such cases exceeded the number of confirmed recurrences. Such instances in which retreatment of study subjects occurred without full culture confirmation had been prospectively designated as recurrences by the study protocol [6].

**Table 2:** Observed and predicted relapse rates in arms of three phase 3 trials of fluoroquinolone-containing regimens.

Study/regimen	Duration	Positive at month 2	Recurrences			
			Evaluable	Observed	Observed	Predicted
**REMox**	*(months)*	*(proportion)*	*(N)*	*(N)*	*(proportion)*	*(proportion)*
2HREZ/4HR	6	0.164	502	12+14	0.052	0.064
2HRMZ/2HRM	4	0.143	503	46+17	0.125	0.136
2ERMZ/2RM	4	0.123	512	64+27	0.178	0.127
**OFLOTUB**						
2HREZ/4HR	6	0.167	676	53	0.078	0.064
2HRGZ/2HRG	4	0.141	687	108	0.157	0.135
**RIFAQUIN**						
2HREZ/4HR	6	0.076	160	4+1	0.031	0.043
2EMRZ/4P_1_M_1_	6	0.053	185	4+1	0.027	0.036
2EMRZ/2P_2_M_2_	4	0.053	163	19+7	0.160	0.086

Evaluable subjects are those who at end-of-treatment have not met other unsatisfactory endpoints. Observed relapse rates are from per-protocol analyses, calculated as the number of subjects meeting the primary definition of recurrence in each trial (REMox: “relapse” + “retreated”; OFLOTUB: unfavorable outcomes at 18 months; RIFAQUIN: “culture confirmed” + “other”) divided by the number of evaluable subjects. Relapse was predicted using a model developed without data from the 3 trials in question, whose variables included total treatment duration and month 2 culture status using solid media [5]. E = ethambutol; G = gatifloxacin; H = isoniazid; M = moxifloxacin; P = rifapentine; R = rifampin; Z = pyrazinamide. Leading numbers in regimens indicate duration in months. Drugs were administered 7 days per week except as indicated by subscripts.

**Fig 1:**
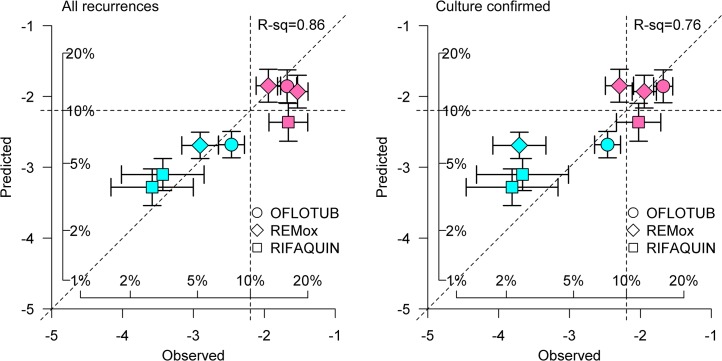
Observed and predicted proportions of subjects with tuberculosis recurrence in the 8 arms of the 3 trials comprising the validation dataset, based on all recurrences (left panel) and only those with full culture confirmation (right panel). Recurrences were predicted using the original mathematical model as reported in reference [5]. Axes indicate logit-transformed recurrence risk; inset scales indicate corresponding percentages. Red symbols indicate 4 month regimens; green symbols indicate 6 month regimens. Error bars indicate 80% confidence intervals (10%-90%). Vertical and horizontal dotted lines indicate recurrence rates of 10% (-2.2 after logit transformation).

The right-most column of Table 2 shows the predicted proportion of patients with recurrence using the model as originally described in 2013. Predictions were based on the proportion culture positive after 2 months of treatment, and the total duration of treatment. Observed and predicted recurrence proportions were highly correlated, with a coefficient (R^2^) of 0.86 and a normalized mean-squared error (NMSE) of 0.04 for the primary analysis of all recurrences (left panel Fig 1). A threshold of 10% (-2.2 on a logit scale) had been proposed in the 2013 publication as the highest recurrence rate that would likely be considered acceptable by tuberculosis control programs. This threshold is indicated by the dotted horizontal and vertical lines (Fig 1). Using this criterion, the original model performed well as a test to predict regimen success, correctly identifying all 4 six-month regimens as satisfactory, and 3 of 4 four-month regimens as unsatisfactory (sensitivity = 100%, specificity = 75%, PPV = 80%, and NPV = 100%). In a secondary analysis that included only recurrences with full culture confirmation, the correlation between observed and predicted recurrence proportions nonetheless remained relatively high (R^2^ = 0.76, NMSE = 0.03). These findings confirm month 2 culture status and treatment duration as predictors of tuberculosis recurrence, and more generally confirm the utility of the mathematical model.

**Fig 2:**
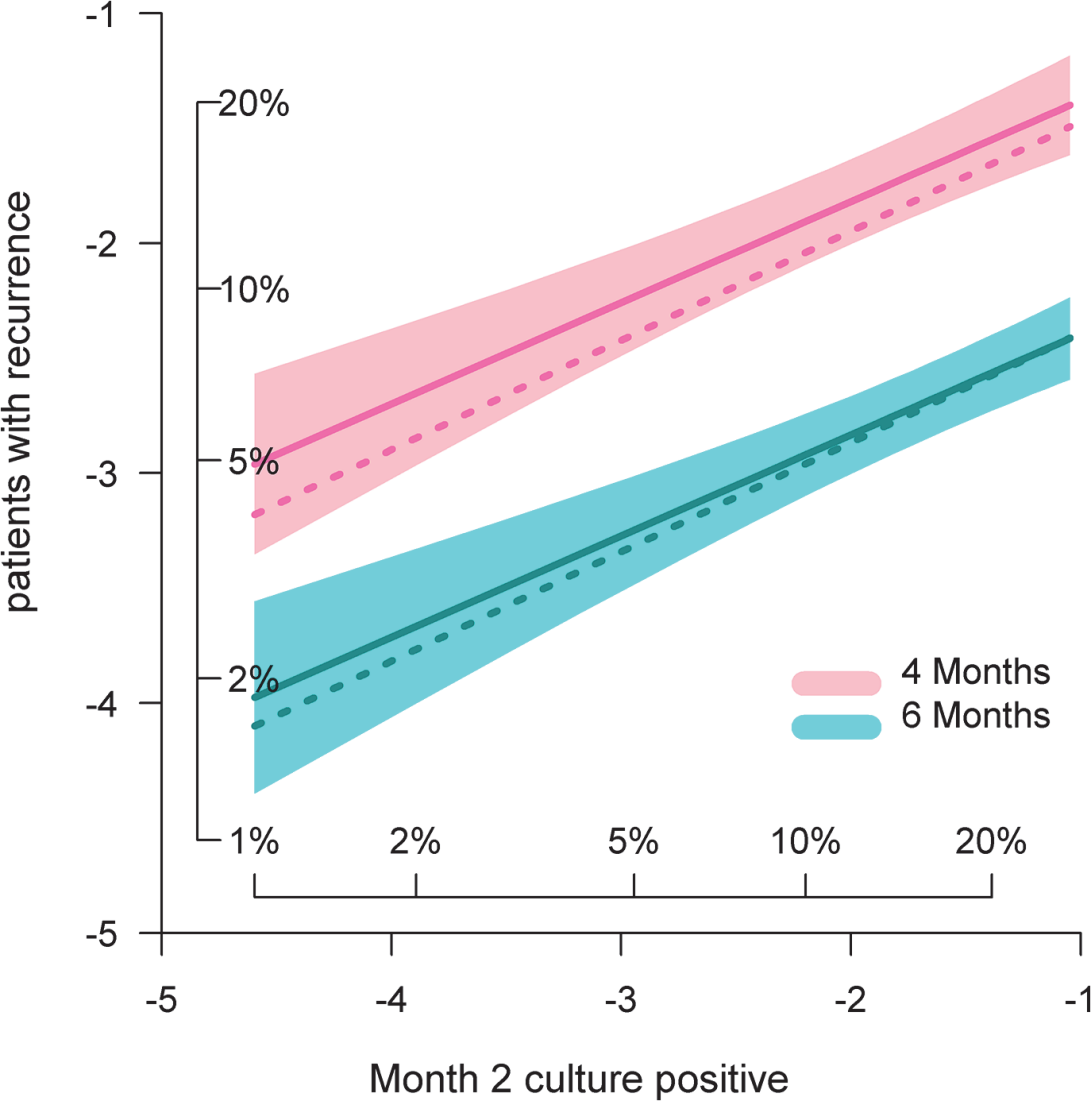
Predicted proportion of patients with recurrence based on the proportion positive after 2 months of treatment, for regimens of 4 and 6 months duration. Axes indicate logit-transformed proportions; inset scales indicate corresponding percentages. Solid and dotted lines indicate updated and original model predictions, respectively. Shading indicates 80% confidence intervals for the updated estimates.

The model was then updated to reflect the full dataset of 27 studies, 66 regimens, and 11181 subjects. The original and revised fitted parameters are shown in Table 3. The revised parameter values for month 2 culture status and duration changed by 8–10%. Supplementary figures are provided showing relationship of relapse to month 2 culture (S1 Fig) and to duration (S2 Fig). Routine diagnostic plots failed to show systematic errors (S3 Fig). The updated model was then used to predict the proportion of tuberculosis recurrences in regimens of 4, 6, and 8 months duration, in relation to month 2 positive proportions ranging from 0.005 to 0.5 (0.5% to 50%). Fig 2 shows predicted values (solid lines) and the 80% CI (shading) based on the revised model. Dotted lines show values predicted by the original model for comparison. The main effect of the revision was to increase to 10% the predicted recurrence rate in the sole 4-month fluoroquinolone regimen that had been incorrectly predicted to yield acceptable results. Table 4 shows corresponding results for the 80% prediction interval (PI) for a hypothetical trial with 680 patients per arm. Parameters yielding a risk of approximately 10% of a relapse rate >10% are indicated in bold. The target month-2 culture positive rate identified by the revised model for a new 4-month regimen remained 1%.

**Table 3:** Parameter estimates of original and revised meta-regression models.

Parameter	Estimate	SE (CV%)	P
**Original model**			
Intercept	2.1471	0.6092 (28.4%)	0.0018
Natural log treatment duration	-2.2670	0.2958 (13.0%)	<0.0001
Logit month 2 culture positive rate	0.4756	0.1063 (22.4%)	<0.0001
**Revised model**			
Intercept	2.5289	0.4931 (19.9%)	<0.0001
Natural log treatment duration	-2.5018	0.2299 (9.3%)	<0.0001
Logit month 2 culture positive rate	0.4399	0.1004 (22.0%)	<0.0001

SE = standard error; CV = coefficient of variation.

**Table 4:** Predicted relapse rates for hypothetical regimens of 4, 6, and 8 months duration.

Treatment duration (mo)	Month 2 positive rate (%)	Predicted relapse rate (%)	80% PI
**4**	**1**	**4.9**	**2.1, 10.9**
4	2	6.6	3.0, 13.8
4	3	7.8	3.7, 15.9
4	4	8.8	4.2, 17.6
4	5	9.7	4.7, 19.0
4	6	10.4	5.1, 20.2
4	8	11.8	5.8, 22.4
4	10	12.9	6.4, 24.3
4	13	14.5	7.3, 26.7
4	18	16.7	8.5, 30.2
4	26	19.8	10.2, 34.8
4	36	23.3	12.2, 39.9
6	1	1.8	0.7, 4.5
6	2	2.5	1.1, 5.7
6	3	3.0	1.3, 6.6
6	4	3.4	1.5, 7.4
6	5	3.7	1.7, 8.0
6	6	4.1	1.9, 8.6
6	8	4.6	2.1, 9.7
**6**	**10**	**5.1**	**2.4, 10.6**
6	13	5.8	2.7, 11.8
6	18	6.8	3.2, 13.6
6	26	8.2	4.0, 16.2
6	36	9.9	4.8, 19.3
8	1	0.9	0.3, 2.4
8	2	1.2	0.5, 3.1
8	3	1.5	0.6, 3.5
8	4	1.7	0.7, 3.9
8	5	1.9	0.8, 4.3
8	6	2.0	0.9, 4.6
8	8	2.3	1.0, 5.1
8	10	2.6	1.1, 5.6
8	13	2.9	1.3, 6.3
8	18	3.4	1.6, 7.3
8	26	4.2	1.9, 8.8
**8**	**36**	**5.1**	**2.4, 10.6**

The prediction interval (PI) indicates uncertainty regarding the predicted relapse rate in a hypothetical study with 680 subjects per arm. Parameters yielding a risk of approximately 10% of a relapse rate >10% are indicated in bold.

An assessment of the updated model was performed using data from the TB Research Unit (TBRU) treatment shortening trial reported by Johnson *et al* in 2009 [9]. In that study, 370 HIV-uninfected adult patients with non-cavitary pulmonary tuberculosis at baseline and negative sputum cultures after 2 months of standard treatment were randomly assigned to receive either 2 or 4 additional months treatment with isoniazid plus rifampin. The study was halted by its safety monitoring board when a difference in relapse risk emerged between the 2 arms. The TBRU trial had not been included in the original meta-regression model. The updated model parameters were used to predict the relapse rates for the 2 arms in the trial. Calculations were performed using a month 2 culture positive proportion of 0.005 (0.5%, the lowest in the dataset), as values of zero are not permitted on a logit scale. As indicated in Table 5, observed relapse rates for both arms fell within their respective prediction intervals.

**Table 5:** Observed and predicted relapse rates in the treatment shortening study of Johnson et al [9].

Arm	Total duration	Relapses/Subjects	Relapse rate	
			Observed	Predicted (80% PI)
2HRZE/2HR	4 months	13/185	7.0%	3.7% (1.4%, 9.4%)
2HRZE/4HR	6 months	3/185	1.6%	1.4% (0.4%, 4.4%)

Predictions were based on updated model parameters, a month 2 culture positive proportion of 0.005 (0.5%), and a sample size of N = 185 per arm. H = isoniazid; R = rifampin; Z = pyrazinamide; E = ethambutol.

## Discussion

The translation of the results of phase 2 trials into phase 3 trials is a major challenge for the clinical development of shorter TB regimens. Phase 2 trials typically assess sputum culture conversion, whereas phase 3 trials assess relapse-free cure. Accordingly, TB regimen developers are keen to understand the quantitative link between these endpoints. The meta-regression model originally reported in 2013 and updated here provides a framework for direct translation of Phase 2 results to Phase 3 outcomes. Using the threshold for recurrence of 10% proposed in the original publication as the highest TB control programs would consider acceptable, the present study found that the model as reported in 2013 correctly predicted all 4 six-month regimens in recent trials as satisfactory, and 3 of 4 four-month regimens as unsatisfactory, based on month 2 culture status and duration. Predicted and observed recurrence rates were highly correlated (R^2^ = 0.86). Updating the fitted model using the full dataset of 11181 patients resulted in only minimal changes to its predictions.

It has been argued that the small sample size and resulting wide confidence intervals of typical phase 2 trials limit their ability to predict treatment shortening [6]. However, 5 prior phase 2 trials of 6 gatifloxacin or moxifloxacin-substituted regimens had reported month 2 culture positive proportions of 8–29% [12–15]. The 2013 model predicted that if administered for only 4 months, all 6 regimens would yield unsatisfactory recurrence rates (10.4–19.4%), consistent with those observed in the 3 phase 3 trials (12.5–17.8%) [5,16]. Thus, in these instances, the reduced sample size of the phase 2 trials did not adversely affect the validity of the predictions.

The validation of mathematical models is often conducted by the random allocation of portions of a single dataset for training and validation. Random allocation increases the likelihood that the 2 portions will be comparable, thereby increasing the likelihood that validation will be successful. However, such an approach poses a risk that the model will not perform well in new populations. The validation and training datasets in the present study differ significantly in several key characteristics with the potential to affect the validity of the model. The finding that the original model accurately predicted outcomes despite significant differences in regimen composition, treatment duration, and geographic region indicates the model is robust and generalizable.

The findings regarding the TBRU treatment shortening study [9] are particularly informative in this context. Lung destruction and cavity formation in tuberculosis are driven by the host immune response [17]. Although patients with overt immunodeficiency were excluded from the TBRU trial, host immune factors were nonetheless most likely responsible for the non-cavitary disease and early culture conversion that were required for enrollment. Despite having been derived solely from studies of TB chemotherapy trials, the model accurately predicted outcomes in the TBRU trial. This indicates a potential role of the model to inform the design of future studies in which host-directed and antimicrobial therapies are combined. The relapse rate in the experimental arm of the TBRU trial (7.0%) was unacceptable only in the context of the unusually low relapse rate in the control arm (1.6%). Had the latter been anticipated, alternative study designs might have been considered.

Potential limitations of the present study arise from the comparison of modern and historic data. Formal definitions of intent-to-treat and per-protocol populations were uncommon in the original dataset, whereas they were specified in advance in all three recent trials. Molecular methods to distinguish tuberculosis recurrence due to relapse from that due to reinfection were not previously available. Additional data will be required from future trials if the risk of true relapse is to be modeled. As in the original model, the prediction intervals remain wide, indicating the contribution of other unmeasured predictors of recurrence risk (such as baseline radiographic extent of disease or sputum mycobacterial burden). Due to limitations in the range of regimen durations available in the present data set and the empiric nature of the model, extrapolating predictions of recurrence for regimens shorter than 4 months carries considerable uncertainty. The longest duration studied in the new Phase 3 trials was 6 months; accordingly, the accuracy of the model for regimens longer than 6 months in duration has not yet been prospectively confirmed. The opportunity to do so may arise as treatment-shortening trials in patients with multi-drug resistant tuberculosis are reported. The accuracy of any early biomarker requires that treatment continues as expected after assessment of the biomarker. This consideration necessitated the exclusion from the 2013 analysis of regimens in which rifampin was administered for the first 2 months but not subsequently, as clinical data indicate rifampin must be continued for the entire duration of treatment for its full effect to be evident [18]. This question must be addressed for each future tuberculosis drug on an individual basis. Finally, month 2 culture status remains a relatively weak predictor of outcomes for individual patients.

The science of pharmacometrics has grown in the pharmaceutical industry over the past 2 decades precisely to prevent costly failures in phase 3 trials by identifying and maximizing the factors necessary for success [19]. One of the techniques that emerged is the use of meta-dose-response and meta-regression analysis to inform drug development decision making. The observations of the present study indicate an important role of the meta-regression model to inform the translation of phase 2 culture conversion results to the design and expected outcomes of future phase 3 tuberculosis clinical trials.

## Supporting Information

S1 Fig Scatter plot of logit 2-mo culture positive rates vs. logit relapse rates. (TIF)

S2 Fig Scatter plot of natural log of treatment duration vs. logit relapse rates. (TIF)

S3 Fig Diagnostic plots of standardized residuals for logit relapse rates. A) Predicted values vs. residuals, B) Histogram of residuals and C) Q-Q plot of residuals.
(TIF)
