# Supplementary figures and images for: Month 2 Culture Status and Treatment Duration as Predictors of Recurrence in Pulmonary Tuberculosis: Model Validation and Update
Source: PLoS One. 2015 Apr 29;10(4):e0125403. doi: 10.1371/journal.pone.0125403 (PMC4414505; doi:10.1371/journal.pone.0125403)

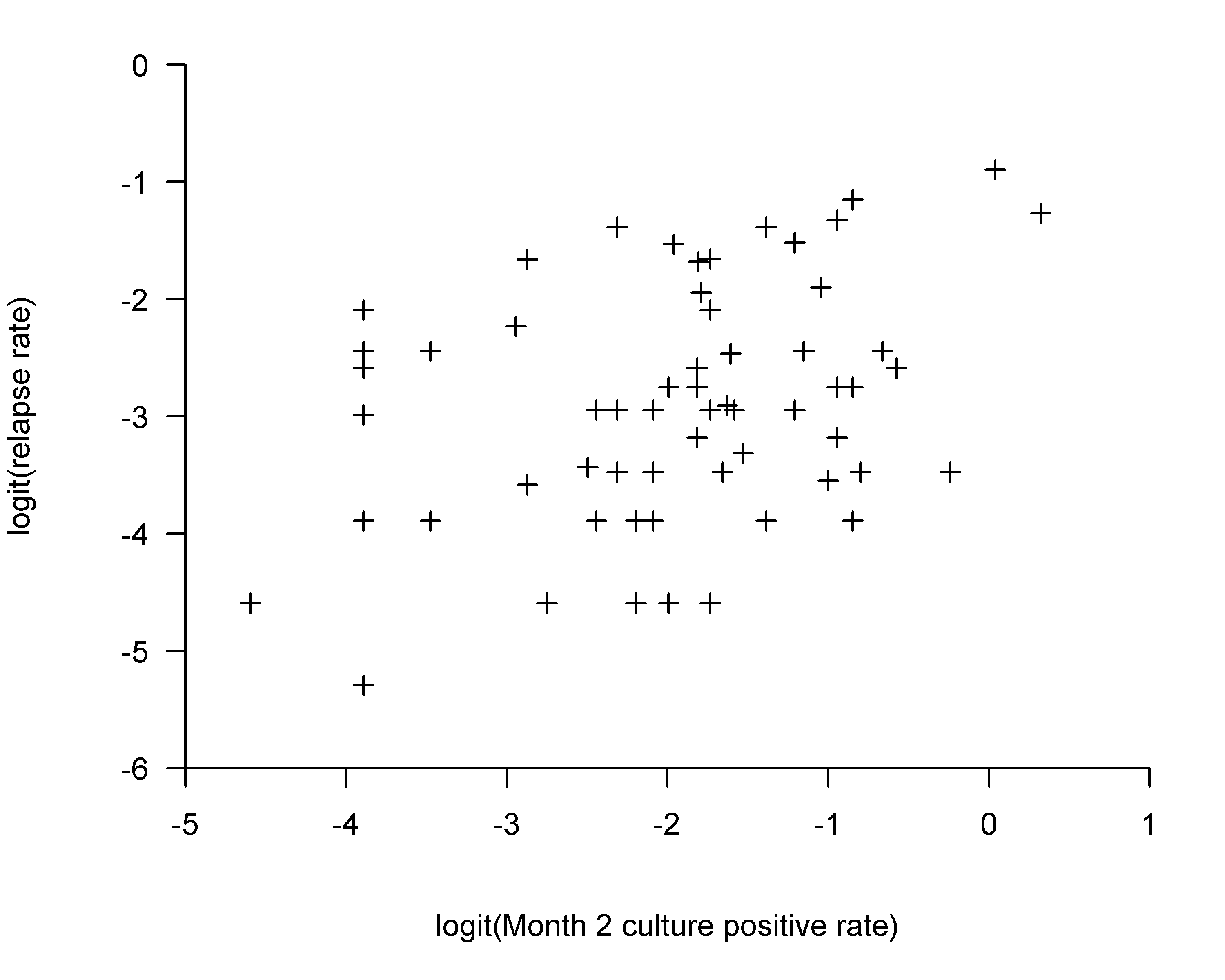

Supplement: S1 Fig — (TIF) [file pone.0125403.s001.tif]

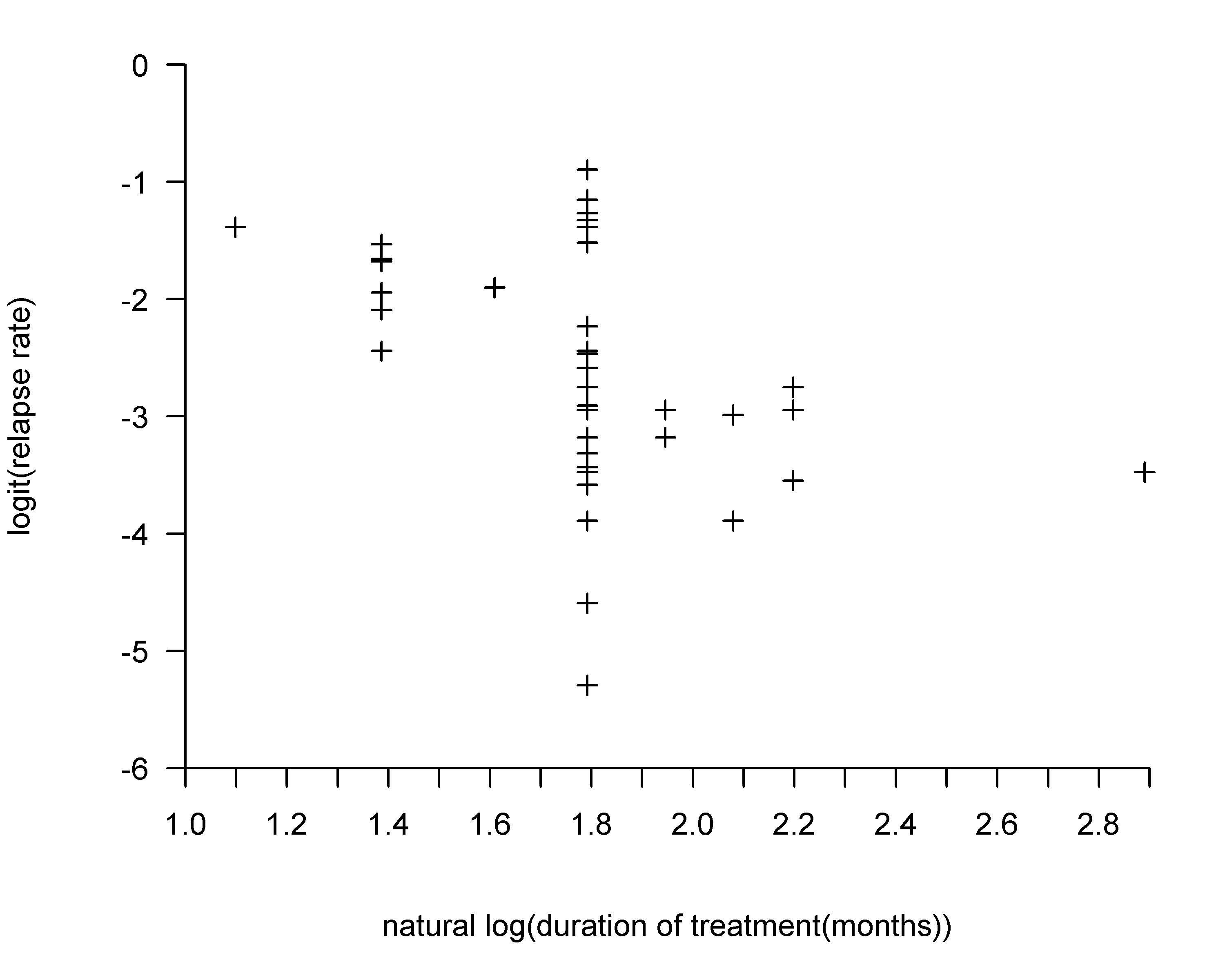

Supplement: S2 Fig — (TIF) [file pone.0125403.s002.tif]

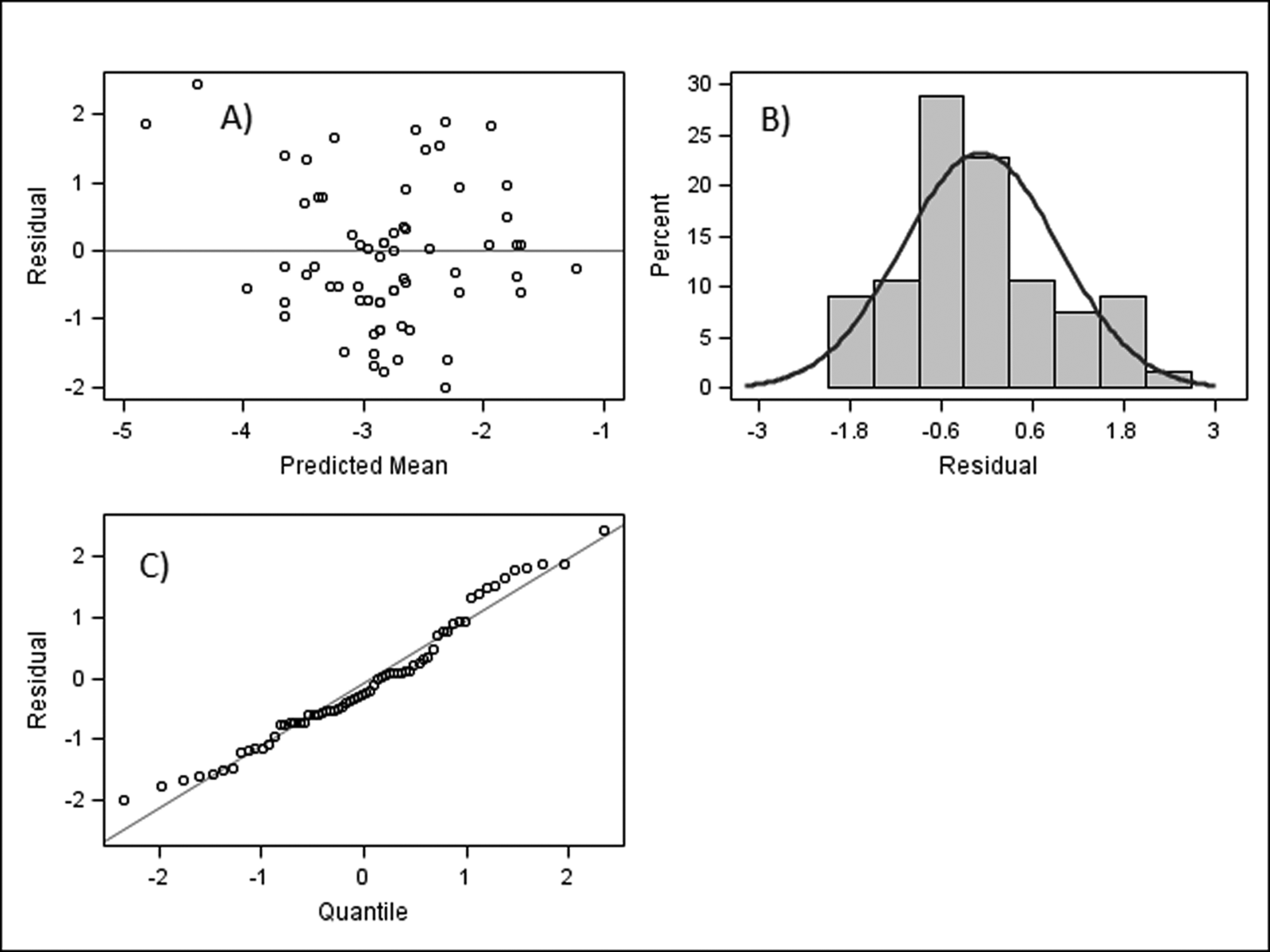

Supplement: S3 Fig — A) Predicted values vs. residuals, B) Histogram of residuals and C) Q-Q plot of residuals. (TIF) [file pone.0125403.s003.tif]
